# Supplementary material for: The Panoptes system uses decoy cyclic nucleotides to defend against phage
Source: Nature. 2025 Oct 1;647(8091):988–96. doi: 10.1038/s41586-025-09557-z (PMC12657218; doi:10.1038/s41586-025-09557-z)

nature portfolio

https://doi.org/10.1038/s41586-025-09557-z

Supplementary information

The Panoptes system uses decoy cyclic nucleotides to defend against phage

In the format provided by the authors and unedited

Supp. Figure 1

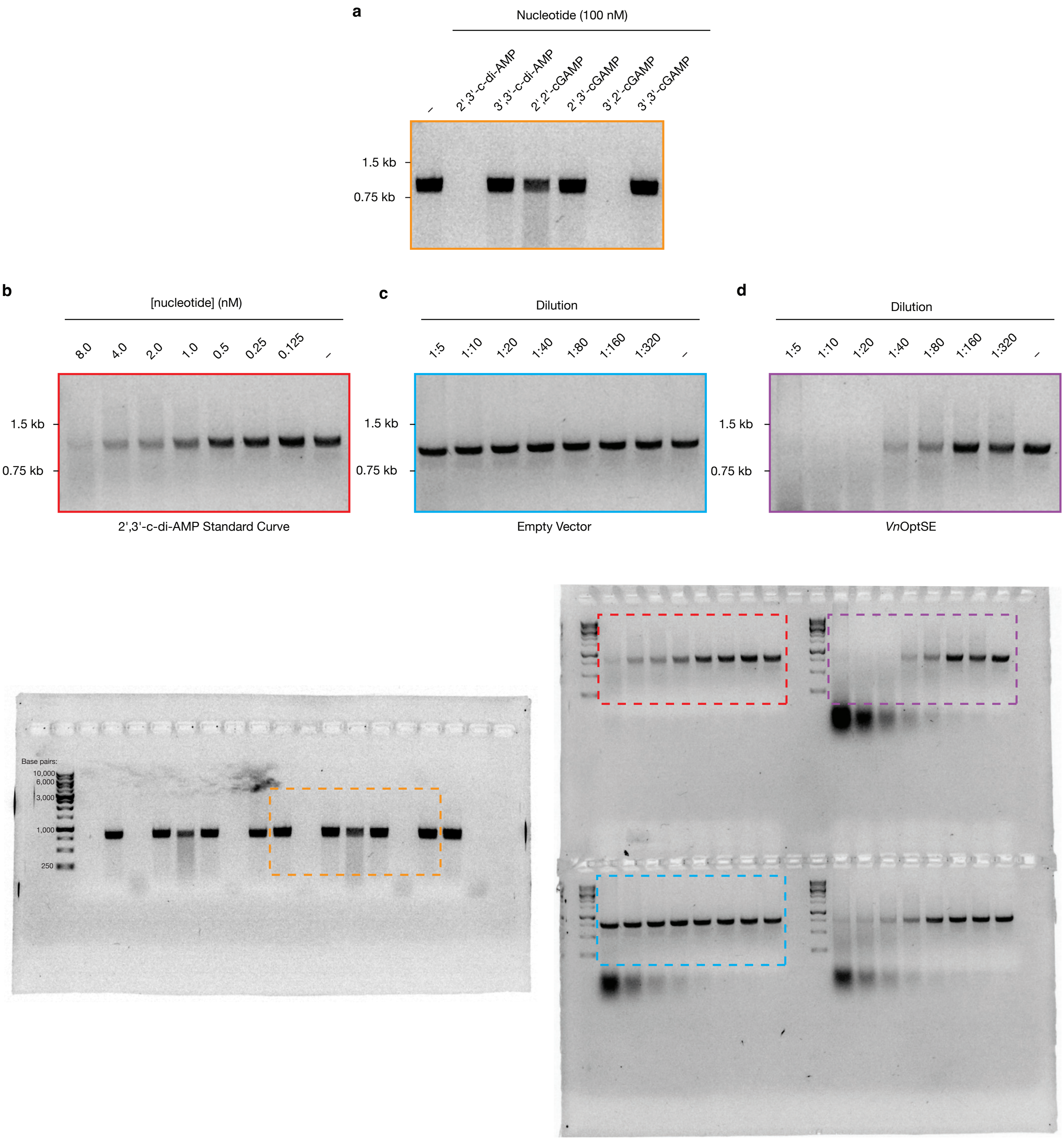

Supplement: Supplementary file 1 — Uncut agarose gel images: Unedited agarose gels corresponding to the figures indicated. [file 41586_2025_9557_MOESM1_ESM.pdf]
